# Do Positive Emotions Make You More Prosocial? Direct and Indirect Effects of an Intervention Program on Prosociality in Colombian Adolescents During Social Isolation Due to COVID-19

**DOI:** 10.3389/fpsyg.2021.710037

**Published:** 2021-08-12

**Authors:** Belén Mesurado, Santiago Resett, Mariana Tezón, Claudia E. Vanney

**Affiliations:** ^1^Instituto de Filosofía, Universidad Austral, Pilar, Argentina; ^2^Consejo Nacional de Investigaciones Científicas y Técnicas (CONICET), Buenos Aires, Argentina; ^3^Grupo de Investigación Psicología, Ciencia y Tecnología, Programa de Psicología, Corporación Universitaria Iberoamericana, Bogotá, Colombia

**Keywords:** adolescents, COVID-19, intervention, prosocial behavior, positive emotion

## Abstract

The objectives of this study are to analyze the efficacy of the Virtual Hero Program during the social isolation due to COVID-19 to increase the positive emotions (joy, gratitude, serenity, personal satisfaction, and sympathy) and prosocial behavior of Colombian adolescents. Additionally, we will analyze whether the Hero program, by directly promoting positive emotional states in adolescents, can predispose them to take prosocial actions toward other people (via an indirect or mediated effect). The final sample of the study comprised 100 participants from the intervention group (*M* age = 13.94, *SD* = 0.97) and 111 from the control group (*M* age = 14.39, *SD* = 0.81). The participants were assigned to the groups using a cluster randomized trial. The positive emotions questionnaire and the Kindness and Generosity subscale of the Values in Action Inventory of Strengths were used to measure the variables. The results indicated that the program increased joy, gratitude, serenity and personal satisfaction but not sympathy of those who participated in the intervention. The promotion of these positive emotions predisposed the Colombian adolescents to act prosocially. Furthermore, the program was also effective in directly promoting prosocial behaviors in the adolescents during social isolation, as observed through a statistically significant difference in the pre- and post-test evaluations between the control and intervention groups. The structure of the intervention brought adolescents closer to social situations to which isolation had limited their access, promoting the importance of closeness and solidarity with others within the complexities of the social confinement context. This study is particularly relevant because interventions with proven effectiveness are necessary to counteract the trauma produced by social isolation in young people throughout the world.

## Introduction

### Beginnings of COVID-19 and Its Characteristics in Colombia

COVID-19, the disease caused by the new coronavirus called SARS-CoV-2, appeared for the first time in Wuhan, China, in late December 2019 (Kamps and Hoffmann, 2020) and generated a global health emergency due to its rapid spread and a lack of knowledge regarding effective treatments (World Health Organization, 2020). More precisely, in December 2019, reports emerged of cases of pneumonia of unknown cause in Wuhan, China, which culminated in the identification of a new coronavirus on January 12, 2020; the virus was named SARS-CoV-2, and the associated disease was named COVID-19 (Li et al., 2020). The virus spread rapidly throughout the world and was declared a pandemic by the World Health Organization (WHO) on March 11, 2020 (World Health Organization, 2020). Many countries adopted unprecedented public health measures to curb its expansion (Mehta et al., 2020), although these measures were somewhat fruitless.

To date, there have been more than 130 million cases of COVID-19, and it has caused more than 2,900,000 deaths worldwide. The United States is the country with the highest number of cumulative cases of COVID-19, with more than 30,000,000 infected patients and 550,000 deaths. In Latin America, Colombia has more than 2,430,000 infections and almost 70,000 deaths (World Health Organization, 2021).

Colombia, like other Latin American countries, has been on alert and expectant about the world situation. The Colombian government issued a decree on March 18, 2020 (Dec. 457), that announced mandatory preventive social isolation for 19 days, with the intention of returning to normal activities in early April 2020. The Colombian Ministry of Education announced that on April 13, 2020, it would announce the actions to be taken in educational institutions. Due to the increase in the number of infections, the Colombian Ministry of National Education decided to extend the mid-year vacation until April 21. At the end of the holidays, the government announced the implementation of virtual education for the entire 2020 school year at the preschool, elementary and higher levels due to the health emergency generated by the pandemic (La Opinión, 2020). In this regard, the national government and the Ministry of National Education in Colombia included recommendations and measures developed by UNICEF (2021) to support the continuity of school activities by developing contingency plans around the closure of schools: i) distance learning: ii) emotional containment of vulnerable youth: iii) pedagogical actions for the prevention of COVID-19.

### Negative Effects of COVID-19 on Adolescent Mental Health

As soon as the pandemic began in April 2020, Van Bavel et al. (2020) published an article aggregating a valuable body of material from the social sciences literature warning about the possible negative impact of the pandemic. These authors discussed research on the perception of an arising threat (e.g., fear of contagion, prejudice and discrimination toward possibly infected people), the impact of the pandemic situation in the social context (e.g., social iniquity, vulnerable populations), the pandemic as a stressor and coping strategies (e.g., physical and social distancing), among others (Van Bavel et al., 2020).

Social distancing, or quarantine, known as a universal non-pharmacological intervention, was considered the best strategy to avoid contagion by most countries worldwide. Since April 2020, almost four billion people in more than 80 nations have been affected by social isolation to prevent the spread of the virus. This measure affected social contact and generated some negative psychosocial effects (Venkatesh and Edirappuli, 2020). Research has shown that isolation gave rise to negative psychosocial and physical correlates in individuals (Pfefferbaum and North, 2020), significantly reduced in-person activities, limited productivity and affected the economy, and simultaneously increased unemployment and general unrest in the population (Hevia and Neumeyer, 2020). Adolescents were not excluded from the negative effects of this isolation (Espada et al., 2020; Stankovska et al., 2020), particularly because this stage of life is a sensitive period in which the need for interpersonal contact is vital (Orben et al., 2020). In this sense, spending long periods without attending school—a space not only for learning but also for socialization—can also be a mental health risk factor in adolescents (Ali et al., 2019). A study on the psychosocial effects of isolation due to COVID-19 in adolescents aged 12 to 18 years from China found that 44% showed depressive symptoms, 37% had anxious symptoms, and 31% had both. Another study developed by Duan et al. (2020) arrived at similar results. Similarly, one study found an increase in depression and anxiety and a decrease in life satisfaction based on measurements taken at two timepoints during isolation, indicating that adolescents are more concerned about government restrictions than about the virus or the disease (Magson et al., 2021). This same study also showed that discomfort was more pronounced in adolescent girls than boys (Magson et al., 2021). A recent review of the psychosocial effects of social isolation in children and adolescents indicated that for adolescents, the main presentations are irritation, nervousness, frustration and boredom (Imran et al., 2020).

It is important to highlight that the effects of social isolation depend on its duration. In this sense, a recent meta-analysis that included 24 studies found that prolonged periods of quarantine—more than 10 days—were associated with reduced mental health, with the major presenting symptoms of post-traumatic stress, avoidance behaviors and anger (Brooks et al., 2020). In addition, this research indicated that prolonged quarantine causes boredom, frustration and fear in the population (Brooks et al., 2020). Additionally, there are long-term or post-quarantine psychosocial effects, such as decreased financial income, stigmatization of people who were infected and even the presence of symptoms of post-traumatic stress. Such effects can seriously threaten the health of adolescents in nations with long periods of isolation, such as Colombia. In Colombia, strict compulsory isolation was extended from March 15 to August 30, 2020, and from September 1, 2020 to the present, and some specific activities were restricted; for example, in-class classes at public schools continue to be canceled, and the circulation of the population remains restricted. For this reason, interventions that promote well-being and positive relationships with others in the context of a health emergency are of great importance to reduce the negative psychosocial effects of isolation. One possible way to reduce the psychosocial costs of social isolation is to increase levels of positive emotions and prosocial behavior to restore and improve adolescents' interpersonal networks.

### Positive Emotion and Prosocial Behavior in Adolescents

Positive emotions are brief, multidimensional responses to changes that people detect in different circumstances of their lives; some of the most frequently studied are joy, gratitude, serenity, personal satisfaction, and sympathy (Fredrickson, 2013; Oros, 2014). Recent theories postulate that emotions can self-perpetuate systems that trigger behaviors that help people maintain or prolong their current states (Kuppens et al., 2010; Wichers, 2014). This can be explained by mood-maintenance theory, which indicates that positive emotions generate prosocial behaviors and, in turn, maintain or restore these emotions to their original levels (Isen and Simmonds, 1978). Prosocial behavior is understood as positive social acts to promote the well-being of others (Brief and Motowidlo, 1986). This construct is associated with important positive psychosocial correlates, such as higher self-esteem and improved interpersonal relationships (Laible et al., 2004; Padilla-Walker and Carlo, 2014).

The relationship between emotions and prosocial behavior is well-established (Hammond and Brownell, 2018). There are some experimental studies that show that promoting emotion in general increases the prosocial behavior of cooperation (i.e., working together for a common goal) (Rand, 2016; Levine et al., 2018; Kvarven et al., 2020) and decreases instrumental harm (Capraro et al., 2019). In fact, a recent metanalysis indicated that people who rely on their emotions more than reason to make decisions tend to be more cooperative (Kvarven et al., 2020). In addition, positive emotions play a central role in the development of prosociality (Hammond and Drummond, 2019) and moral standards (Hart and Matsuba, 2007; Tracy and Robins, 2007). One positive emotion is joy, which refers to a general state of fun and rejoicing (Lazarus, 2006) involving a positive affect and a positive cognitive evaluation of one's life (Veenhoven, 2010). Previous studies indicate that joy—or happiness—creates a domino effect, increasing positive emotional states and causing adolescents to act in a more prosocial way (Erreygers et al., 2019). Gratitude is another positive emotion of great social relevance that is experienced when a positive benefit is intentionally granted by another individual and is not achieved through one's own effort (Emmons et al., 2003). Thus, gratitude is a positive experience that implies a generalized tendency to recognize the commitment of others to one's own benefit and to respond to this with gratitude (McCullough et al., 2002). According to McCullough et al. (2002), it is likely that people with a higher level of gratitude more strongly perceive the social support they receive from others. You et al. (2020) found that gratitude directly predicted prosocial behavior in adolescents. In addition, serenity is a feeling of peace and trust that can be experienced independently of external events and involves inner peace, even in the face of adverse events (Connors et al., 1999). Previous research has found that those who perceive themselves as more serene show higher levels of prosocial behaviors (Connors et al., 1999). Finally, sympathy constitutes the affective component of empathy and is the ability to tune into the emotions of others and the inclination to help (Oros, 2014). Padilla-Walker et al. (2015) found that sympathy mediates the relationship between friendship connections and prosocial behavior in adolescents. On the other hand, a recent investigation by García-Vázquez et al. (2020) evaluated the role of different positive emotions (forgiveness, gratitude and happiness) in predicting whether adolescents would engage in prosocial behaviors to stop bullying. The results indicated that the three emotions had a positive direct effect on the development of prosocial behaviors to stop bullying or help victims.

Given that the abovementioned studies show that the presence of positive emotions predisposes adolescents to engage in prosocial actions toward others, we thus propose in this study to promote the positive emotional states of adolescents with the intention of stimulating prosocial behaviors. Because adolescents are isolated by the pandemic situation, it seems appropriate to use technological interventions that allow us to reach their homes with the intention of mitigating psychological distress and promoting their well-being. Indeed, Van Bavel et al. (2020) suggest that online interactions could be a means by which to develop a sense of connection with others, thereby improving psychological well-being; this phenomenon is especially likely to occur if the technological tool is dynamic and synchronous (p. 466). In this way, the Hero Program can be useful to reduce the adverse psychosocial effects of isolation.

### The Hero Program

The Hero Program was developed and tested by Mesurado et al. (2019b) and targets at Spanish-speaking adolescents between 12 and 15 years of age. It is a short, online program composed of five intervention sessions. The first session seeks to stimulate the recognition of emotions and empathy; the second session seeks to stimulate a specific positive emotion (gratitude); the third session aims to stimulate other types of positive emotions, such as joy and serenity; the fourth is aimed at promoting forgiveness; and the fifth stimulates empathy toward people who need help (prosocial behavior) (Mesurado et al., 2020). According to Mesurado et al. (2019b), the variables empathy, gratitude, positive emotions and forgiveness were chosen to be part of the program because they can be taught and because there is empirical evidence of their predictive effects on prosociality (Mesurado et al., 2019b). The program was created and tested in Latin American adolescents from Argentina and Uruguay (Mesurado et al., 2019b, 2020). Each intervention lasts ~40 min to an h. The program demonstrates high levels of acceptance among adolescents and is effective in the development of prosocial behaviors (Mesurado et al., 2019a,b, 2020). In addition, it was found to be effective for promoting empathy and positive emotions, and its effects are maintained for as long as three months after the end of the intervention (Mesurado et al., 2020). Consequently, Hero seems to be a promising program for promoting positive adolescent development, mainly due to the frequent use of technology by young people, which may have increased due to isolation.

Given the importance of positive emotions and prosociality to the psychological well-being of adolescents, we plan to implement a virtual program—such as Hero—to help improve the emotional state of Colombian adolescents during a complex health and social situation, such as the social isolation generated by the pandemic. It is plausible that COVID-19, with its isolation measures, is the greatest collective trauma that most human beings have experienced (Rosenfeld et al., 2021). Research indicates that collective trauma from COVID-19 may foster generous behavior (Lotti, 2020), while other research indicates that such trauma may undermine prosocial tendencies and actions aimed at helping others (Brañas-Garza et al., 2020). Indeed, Lotti (2020) found that pandemic-related worry had a positive effect on donations. Conversely, Brañas-Garza et al. (2020) carried out an online experiment for 6 days at a time when the number of deaths due the pandemic in Spain rose extremely quickly; their results indicated that generosity decreased significantly during this period, especially in elderly participants. This may be because the increasing number of deaths and the fear of getting sick have caused a reduction in empathy and compassion (Cameron and Payne, 2011; Västfjäll et al., 2014).

Based on the above, the objectives of this study are to analyze the effectiveness of the Hero Program for improving positive emotions (joy, gratitude, serenity, personal satisfaction, and sympathy) and prosocial behavior in Colombian adolescents during social isolation due to COVID-19. Additionally, we will analyze whether the Hero program, by directly promoting positive emotional states in adolescents, can in turn predispose them to perform prosocial actions toward other people (i.e., its indirect or mediated effect).

Previous research has shown differences in emotional experiences between genders (Brebner, 2003; Chaplin and Aldao, 2013). With regard to prosocial behavior, the discussion is still open, especially in the case of cooperation: one meta-analysis found no gender differences (Balliet et al., 2011), while another found gender differences (Rand, 2017). In terms of altruistic behavior (i.e., prosocial behavior aimed at selflessly seeking the welfare of others), four meta-analyses have been performed, all of which agree that women tend to me more altruistic than men (Engel, 2011; Rand et al., 2016; Brañas-Garza et al., 2018; Xiao et al., 2019). Based on this background, we will control the gender of the participants in the analyses. Likewise, the age of the adolescents will be controlled because previous longitudinal studies have shown changes over time (Van der Graaff et al., 2018).

This paper make at least three novel contributions to the current research developed using the Hero Program: (1) evaluating for the first time the effectiveness of the Hero Program in adolescents in situations of social and physical isolation, (2) applying the research for the first time in a new country in Latin America, Colombia, thus increasing the evidence of its efficacy in different cultural contexts, and (3) studying the mediating effect of positive emotions between the interventions and prosocial behavior, a feature not previously analyzed.

## Materials and Methods

### Design

To analyze the effectiveness of the Hero program in Colombian adolescents, a pre-test and post-test research design with a control group was used. The allocation of participants to the intervention group and the control group waiting list was performed using a cluster randomized trial (Campbell et al., 2004). Students from three Colombian educational institutions participated; students from five courses were randomly included in the control group, and students from five other courses were included in the experimental group. The research protocol of this study was approved by the Comité Institucional de Evaluación of the Facultad de Ciencias Biomédicas of Universidad Austral [CIE N° P 20-058], and it was ratified by the ethics committee of the Ibero-American University Corporation.

### Participants

**Figure 1:**
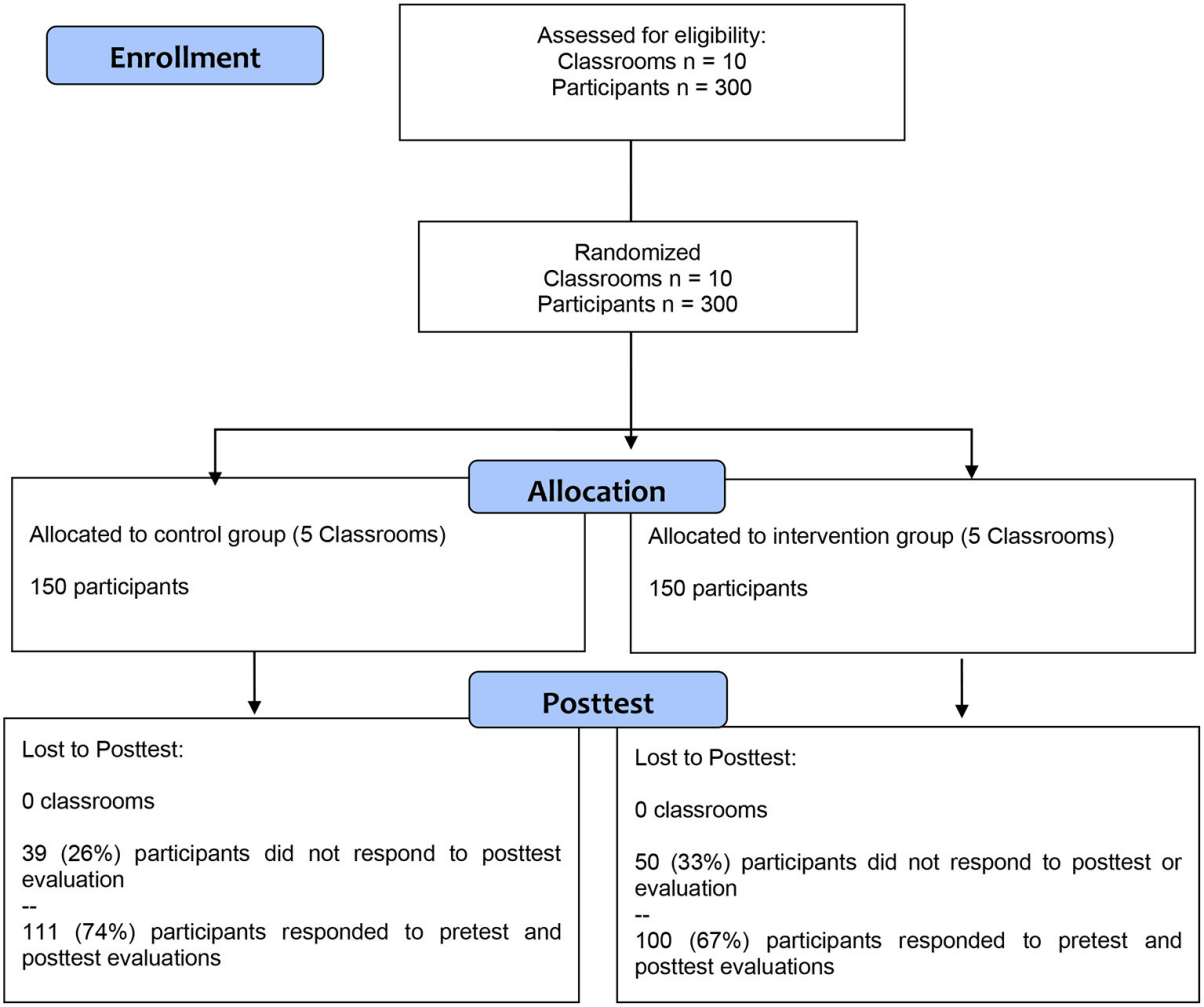
Participant flow chart.

The study comprised 300 participants of both genders between 12 and 15 years (*M*_age_ = 14.09, *SD* = 1.09) from the city of Cartagena de Indias, Colombia. Thirty-three percent of the participants in the intervention group and 26% of the participants in the control group left the study. Figure 1 shows the flow diagram of the participants according to the guidelines of Consolidated Standards of Reporting Trials (CONSORT). The final sample of the study was composed of 100 participants from the intervention group (*M*_age_ = 13.94, *SD* = 0.97) and 111 from the control group (*M*_age_ = 14.39, *SD* = 0.81), and 42% of the sample were female. Regarding the educational level of the participants' fathers, as reported by the participants, 12.9% of the fathers completed primary education, 31.4% completed secondary education, 30% completed tertiary or university education and 25.7% of the adolescents said they were unaware of their fathers' maximum education level. Regarding their mothers, the adolescents reported that 10.5% of their mothers had completed primary education, 31% had completed secondary education, 42.4% had completed tertiary or university studies, and 16.2% of the adolescents reported not knowing their mothers' maximum education level.

### Procedure

A researcher from the project who was based in the city of Cartagena de Indias, Colombia, contacted secondary schools in the city, and the selection was intentional. Subsequently, meetings were held with the directors of the schools, at which the research project was presented, and the characteristics of the virtual intervention program were shown. The directors who were interested in implementing the program with the students at their educational institutions allowed us to organize meetings with the secondary school coordinators (teachers who organize the activity of the teachers in charge of the subjects taught at the school) and the teachers in charge of teaching “Ethics and Values” and “Project and Life.” At these meetings, the program description, the technical requirements for implementation and the possible intervention schedule were presented again. Finally, meetings were held with the parents or guardians of the students who would participate in the research project to provide them with the same information. At the end of the meeting, the parents or guardians were given informed consent forms for review that guaranteed the protection of the data collected in the investigation, clarified that the participation of the adolescents was voluntary and stated that the adolescents must also provide their consent. The parents were given seven days to read the consent carefully, and those who were interested in having their children participate in the study were asked to return the signed form to the school. The research was implemented within the framework of activities developed in the “Ethics and Values” or “Project and Life” courses, depending on the institution. The adolescents in the intervention group virtually attended seven weekly meetings from their homes (a pre-test evaluation, five intervention sessions and a post-test evaluation), while those in the control group attended two meetings (pre-test and post-test evaluations). Each encounter lasted 45 min to one h. The meetings were held synchronously through the Google Meet platform, and each session was coordinated by a teacher from the educational institution and a researcher from the research team. The program was implemented from March 30 to July 30, 2020.

### Instruments

#### Positive Emotions

Five types of positive emotions, joy, gratitude, serenity, personal satisfaction, and sympathy, were evaluated using the Argentine positive emotions questionnaire by Oñate and Mesurado (2021). This questionnaire is an adaptation of the questionnaire of positive emotions for children by Oros (2014) for adolescents. The questionnaire consists of 23 statements; the adolescent must indicate the frequency with which he or she experiences these emotions using a response scale of 1 (never), 2 (sometimes) or 3 (always). To obtain the score, the responses to the items in each subscale must be averaged. Below example items for each subscale with their respective internal consistency index in the pre-test and post-test evaluation: joy, e.g., “I am very happy” (McDonald's coefficient omega 0.72 and 0.80); gratitude; e.g., “I like to thank people” (McDonald's coefficient omega 0.77 and 0.84); serenity, e.g., “Most days I feel at peace” (McDonald's coefficient omega 0.81 and 0.82); personal satisfaction, e.g., “I feel that I am important” (McDonald's coefficient omega 0.84 and 0.81); and sympathy, e.g., “I feel very bad if I see someone get hurt” (negative item, scored inversely) (McDonald's coefficient omega 0.76 and 0.88).

#### Prosocial Behavior

To evaluate prosociality, the Kindness and Generosity subscale of the Values in Action Inventory of Strengths by Peterson and Seligman (2004) was used. This subscale was validated in Spanish by Mesurado et al. (2019c). The scale consists of nine statements (e.g., “I enjoy being kind to others”). Adolescents should indicate the degree to which each of the statements represents them using a scale from 1 (not like me at all) to 5 (very much like me). To obtain the score, the answers given for each item must be averaged. McDonald's coefficient omega, which evaluates the internal consistency of the items, was 0.84 for the pre-test evaluation and 0.93 for the post-test evaluation.

At the end of the post-test evaluation, the adolescents in the intervention group were asked to indicate specific ways in which the program had impacted their daily lives. It was not mandatory for adolescents to complete this step. This question was asked with the intention of collecting qualitative information to show the impact of the program on adolescents and to better understand the statistical results.

### Statistical Procedure

The statistical package SPSS 24 was used to calculate the mean and standard deviation of all the data obtained from the pre-test and post-test measurements of the control group and the intervention group. Moreover, SPSS 24 was used also to carried out chi-squared tests, ANOVA, and MANOVA for the preliminary analyses.

In addition, to analyze the objectives of the study, the statistical program MPLUS 8.5 by Muthén and Muthén (2017) was utilized. Five different models were analyzed using the analysis of covariance (ANCOVA) proposed by Valente and MacKinnon (2017), a statistical technique that has been used in previous research to evaluate the effectiveness of intervention programs (Luengo Kanacri et al., 2020). ANCOVA is conducive to analyzing the direct effect of interventions on the promotion of two variables using a pre-test and post-test design. It also allows the measurement effect of one of the variables for promoting another linked variable to be analyzed. Additionally, it analyzed whether the program promotes a specific type of positive emotion (namely, joy, gratitude, serenity, personal satisfaction, and sympathy) and examined via mediation analysis whether this in turn promotes the development of prosocial actions toward other people.

**Figure 2:**
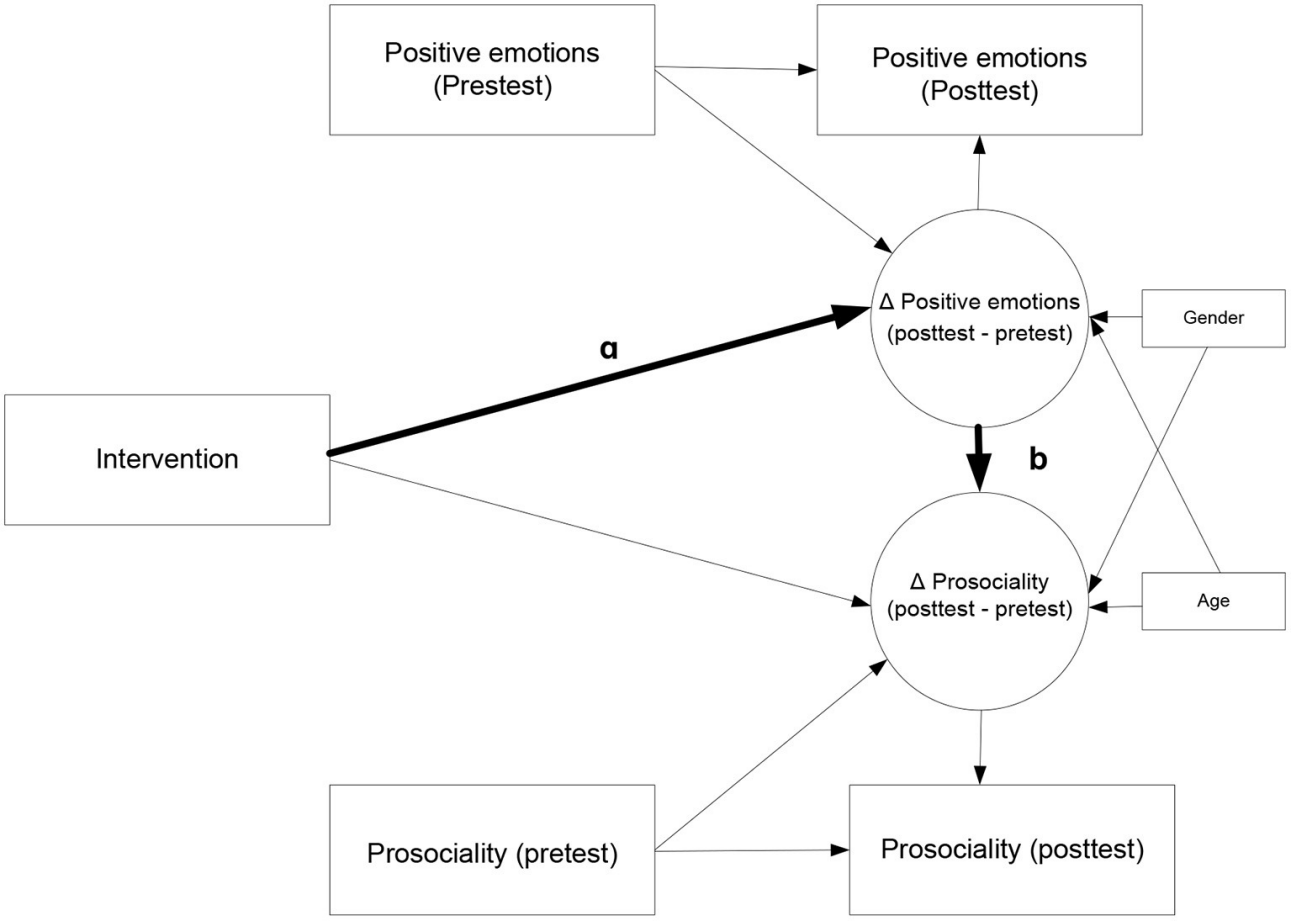
Models tested. Model 1 used joy, Model 2 used gratitude, Model 3 used serenity, Model 4 used satisfaction, and Model 5 used sympathy as positive emotion mediating variable.

This technique allows adjustment by the pre-test of the mediating variable (in this case, positive emotion: joy, gratitude, serenity, personal satisfaction, and sympathy) and the outcome variable (in this case, prosociality). The pre-test evaluation of each positive emotion and prosociality were used in estimating the mediated effect of intervention on prosociality through positive emotions at the post-test evaluation, as suggested by Valente and MacKinnon (2017, p. 430). Specifically, in this article, five different models were run to analyze the effectiveness of the Hero Program for directly promoting five types of positive emotions (model 1: joy, model 2: gratitude, model 3: serenity, model 4: personal satisfaction, and model 5: sympathy) and prosocial behavior (see Figure 2). In all models, the influence of participant gender and age was controlled. Finally, the missing-at-random (MAR) method was used to impute the missing data in each of the models studied (Little et al., 2014).

## Results

### Preliminary Analyses

Table 1 shows the descriptive data (mean and standard deviation) of each of the variables evaluated in the pre- and post-test evaluations of the participants in the control group and the intervention. The participants in the control group and the intervention group were compared in terms of gender, age, father's and mother's educational levels, and their baseline measurements of prosociality and positive emotions (i.e., pre-test evaluation). Three chi-squared tests were used to test for differences between the groups with respect to gender and father's and mother's educational levels. Two one-way ANOVAs were used to test age and prosociality differences, respectively. Finally, a MANOVA was used to test for differences between groups in positive emotions in the pre-test evaluation. The results indicated that the control and intervention groups were equivalent in terms of gender [chi (1) = 2.36, p = 0.12], father's educational level [chi (2) = 0.70, *p* = 0.71], mother's educational level [chi (2) = 3.20, *p* = 0.20], prosociality [*F*_(1, 209)_ = 0.01, *p* = 1.0], and positive emotions [Hotelling's trace *F*_(5, 204)_ = 1.62, *p* = 0.16]. The participants in the control group were older than the participants in the intervention group, *F*_(1, 209)_ = 13.47, *p* < 0.001. Consequently, age was used as a control variable.

**Table 1:** Means and standard deviations of positive emotions (joy, gratitude, serenity, personal satisfaction, and sympathy) and prosocial behavior in the pre-test and post-test evaluations.

**Variables**	**Control Group**				**Intervention Group**			
	**Pre-test**		**Post-test**		**Pre**-**test**		**Post-test**	
	**M**	**SD**	**M**	**SD**	**M**	**SD**	**M**	**SD**
Joy	2.70	0.42	2.66	0.45	2.66	0.38	2.79	0.31
Gratitude	2.76	0.32	2.71	0.37	2.78	0.31	2.84	0.32
Serenity	2.52	0.46	2.57	0.45	2.55	0.39	2.73	0.39
Personal satisfaction	2.63	0.56	2.60	0.57	2.71	0.44	2.78	0.40
Sympathy	2.39	0.52	2.54	0.51	2.30	0.55	2.60	0.46
PB toward strangers	3.21	0.76	3.52	0.84	3.21	0.68	3.93	0.92

### Effects of the Intervention on the Change in Prosociality Scores Using the Positive Emotion Joy as a Mediator (Model 1)

The results indicate that the participants in the intervention group increased their joy (*b* = 0.14, *p* < *0.0*1, 95% CI [0.04 to 0.24]; Cohen's *d* = 0.35, CI [0.07 to 0.62]) and their prosocial behavior toward others (*b* = 0.36, *p* = 0.01, 95% CI [0.14 to 0.60]; Cohen's *d* = 0.37, CI [0.09 to 0.64]) when the intervention ended. Moreover, the findings indicate that the online intervention promoted prosocial behavior toward others through joy (*b* = 0.08, *p* < *0.0*1, CI [0.02 to 0.14]). These findings suggest that the Hero intervention promoted joy, which in turn increased prosocial behavior. These results show that joy partially mediated the effect of the Hero Program on increasing prosocial behavior. Finally, gender and age did not affect the changes in the joy and prosociality scores. This model explained 31% of the joy (*R*^2^ coefficient = 0.31) and 32% of the prosociality (*R*^2^ coefficient = 0.32) (Table 2).

**Table 2:** Hero Program's effect on change scores (Δ) of positive emotions (joy, gratitude, serenity, personal satisfaction, and sympathy) and prosocial behavior.

	**ΔJoy**			**ΔProsocial Behavior**		
**Model 1**	***b***	**SE**	**R^**2**^**	***b***	**SE**	**R^**2**^**
Intervention	0.14^**^	0.05	0.31^***^	0.36^**^	0.12	0.32^***^
Gender	−0.01	0.05		−0.02	0.12	
Age	−0.01	0.02		0.04	0.05	
Pre-test scores	−0.56^***^	0.09		−0.60^***^	0.09	
Mediation				0.08^**^	0.03	
	**ΔGratitude**			**ΔProsocial Behavior**		
**Model 2**	***b***	**SE**	**R** ^**2**^	***b***	**SE**	**R** ^**2**^
Intervention	0.14^**^	0.05	0.31^***^	0.39^***^	0.12	0.32^***^
Gender	0.01	0.05		0.01	0.12	
Age	–0.01	0.05		0.05	0.05	
Pre-test scores	–0.66^***^	0.09		–0.60^***^	0.09	
Mediation				0.07^*^	0.03	
	**ΔSerenity**			**ΔProsocial Behavior**		
**Model 3**	***b***	**SE**	**R** ^**2**^	***b***	**SE**	**R** ^**2**^
Intervention	0.14^**^	0.06	0.35^***^	0.41^***^	0.12	0.31^***^
Gender	–0.07	0.06		–0.01	0.12	
Age	–0.01	0.03		0.04	0.12	
Pre-test scores	–0.66^***^	0.07		–0.62^***^	0.09	
Mediation				0.05^*^	0.02	
	**ΔPersonal Satisfaction**			**ΔProsocial Behavior**		
**Model 4**	***b***	**SE**	**R** ^**2**^	***b***	**SE**	**R** ^**2**^
Intervention	0.15^*^	0.07	0.28^***^	0.40^***^	0.12	0.34^***^
Gender	0.02	0.07		–0.03	0.12	
Age	0.01	0.06		0.03	0.05	
Pre-test scores	–0.53^***^	0.08		−0.60^***^	0.09	
Mediation				0.07^*^	0.03	
	**ΔSympathy**			**ΔPB_E**		
**Model 5**	***b***	**SE**	**R** ^**2**^	***b***	**SE**	**R** ^**2**^
Intervention	0.10	0.07	-	0.39^***^	0.12	0.34^***^
Gender	0.01	0.06		0.07	0.12	
Age	–0.01	0.03		0.05	0.05	
Pre-test scores	–0.69^***^	0.07		–0.58^***^	0.09	
Mediation				0.04	0.03	

*Δ change scores of the variables indicated. The unstandardized b-coefficients were calculated*,

*** *p < 0.001*,

** *p < 0.01*,

* *p < 0.05*.

### Effects of the Intervention on the Change in Prosociality Scores Using the Positive Emotion Gratitude as a Mediator (Model 2)

The results indicate that the participants in the intervention group increased their gratitude (*b* = 0.14, *p* < *0.0*1, 95% CI [0.04 to 0.23]; Cohen's *d* = 0.35, CI [0.07 to 0.63]), and their prosocial behavior (*b* = 0.39, *p* = 0.001, 95% CI [0.17 to 0.62]; Cohen's *d* = 0.41, CI [0.13 to 0.69]) at the end of the intervention. Moreover, the finding indicates that the online intervention promoted prosocial behavior through gratitude (*b* = 0.07, *p* < *0.0*5, CI [0.02 to 0.13]). These findings suggest that the Hero intervention promoted gratitude, which in turn increased prosocial behavior. These results show that gratitude partially mediated the effect of the Hero Program on increasing prosociality. Finally, gender and age did not affect the change in gratitude and prosociality scores. This model explained 31% of the gratitude (*R*^2^ coefficient = 0.31) and 32% of the prosociality (*R*^2^ coefficient = 0.32) (Table 2).

### Effects of the Intervention on the Change in Prosociality Scores Using the Positive Emotion Serenity as a Mediator (Model 3)

The results indicate that the participants in the intervention group increased their serenity (*b* = 0.14, *p* < *0.0*1, 95% CI [0.03 to 0.25]; Cohen's *d* = 0.28, CI [0.01 to 0.56]), and their prosocial behavior (*b* = 0.41, *p* = 0.001, 95% CI [0.17 to 0.65]; Cohen's *d* = 0.43, CI [0.15 to 0.71]) at the end of the intervention. Moreover, the finding indicates that the online intervention promoted prosocial behavior through serenity (*b* = 0.05, *p* < *0.0*5, CI [0.01 to 0.10]). These findings suggest that the Hero intervention promoted serenity, which in turn increased prosocial behavior. These results show that serenity partially mediated the effect of the Hero Program on increasing prosocial behavior. Finally, gender and age did not affect the changes in serenity and prosociality scores. This model explained 35% of the serenity (*R*^2^ coefficient = 0.35) and 31% of the prosociality (*R*^2^ coefficient = 0.31) (Table 2).

### Effects of the Intervention on the Change in Prosociality Scores Using the Positive Emotion Personal Satisfaction as a Mediator (Model 4)

The results indicate that the participants in the intervention group increased their personal satisfaction (*b* = 0.15, *p* = *0.0*2, 95% CI [0.02 to 0.28]; Cohen's *d* = 0.30, CI [0.03 to 0.58]) and their prosocial behavior (*b* = 0.40, *p* = 0.001, 95% CI [0.18 to 0.64]; Cohen's *d* = 0.41, CI [0.13 to 0.69]) at the end of the intervention. Moreover, the finding indicates that the online intervention promoted prosocial behavior through personal satisfaction (*b* = 0.07, *p* = *0.0*2, CI [0.01 to 0.13]). These findings suggest that the Hero intervention promoted personal satisfaction, which in turn increased prosocial behavior. These results show that personal satisfaction partially mediated the effect of the Hero Program on increasing prosocial behavior. Finally, gender and age did not affect the changes in the personal satisfaction and prosociality scores. This model explained 28% of the personal satisfaction (*R*^2^ coefficient = 0.28) and 34% of the prosociality (*R*^2^ coefficient = 0.34) (Table 2).

### Effects of the Intervention on the Change Prosociality Scores Using the Positive Emotion Sympathy as a Mediator (Model 5)

The findings indicate that the participants in the intervention group increased their prosociality (*b* = 0.39, *p* < *0.0*01, 95% CI [0.17 to 0.62]; Cohen's *d* = 0.41, 95% CI [0.13 to 0.68]) but did not increase their sympathy level (*b* = 0.10, *p* = 0.15, 95% CI [−0.04 to 0.23]), at the end of the intervention. Moreover, the results show that the online intervention did not have an indirect effect on prosocial behavior through sympathy (*b* = 0.04, *p* = 0.17, 95% CI [−0.02 to 0.11]). However, the study could be inadequately designed to detect the effect of fostering sympathy vs. the pre-test evaluation due to insufficient statistical power. Finally, gender and age did not affect the changes in personal sympathy and prosociality scores. This model explained 34% of the prosociality (*R*^2^ coefficient = 0.34) (Table 2).

## Discussion

With the appearance of the new disease COVID-19, the world population has experienced an outbreak of such magnitude that the health situation was decreed a pandemic. Colombia mandated confinement of its entire population to their homes from March 15 to August 30, 2020; in-person classes were suspended, and social gatherings were limited. Currently, isolation continues selectively, but schools, for the most part, have not resumed in-person attendance. This isolation has had negative psychological impacts on the adolescent population because their socialization depends on interactions with the peer group and because social behavior is a fundamental component of this stage of development (Hawryluck et al., 2004; Dong and Bouey, 2020; Riiser et al., 2020; Tang et al., 2020). In this sense, peer socialization is a context of utmost importance for the development of adolescents' identity and for their emotional and cognitive development, among other aspects. This social situation has prompted mental health professionals to focus on the emotional and behavioral consequences of social isolation measures (Idoiaga et al., 2020). To this end, it has become necessary to develop interventions that prevent the psychological consequences of isolation, which affect the social and emotional sphere of adolescents in general.

Several studies argue that both positive emotions and prosociality tend to prevent antisocial, violent or aggressive behaviors, improving social relationships in diverse and adverse situations (Romersi et al., 2011). Therefore, interventions that promote both positive emotionality and prosocial behavior in adolescents can contribute to comprehensive, healthy and positive development, promoting the social and emotional skills to counteract problems that can affect their interpersonal relationships (Lam, 2012; Caprara et al., 2015; Mesurado et al., 2019b,d).

The objective of this study was to examine the effectiveness of the Hero Program for directly promoting positive emotions and prosocial behaviors in Colombian adolescents during isolation. Specifically, the program focused on stimulating five positive emotions: joy, gratitude, serenity, personal satisfaction, and sympathy. Additionally, the mediating effect of positive emotions in promoting prosociality was studied.

The effects of the program on the promotion of different types of positive emotions are discussed below, followed by a discussion of the mediating effect of each of the positive emotions on the promotion of prosociality. Finally, the direct effect of the program on the promotion of adolescent prosociality is discussed.

The results indicated that the Hero Program directly promoted *joy;* the adolescents who participated in the intervention showed a statistically significant increased this positive emotion and exhibited a general state of contentment and fun after the intervention. This was observed in the participants' statements that the program was “a lot of fun” and that they noticed that their “mood was better weeks after performing the exercises proposed by the program.” The majority of the adolescents affirmed at the end of the process that the activity made them feel at ease and happy. Furthermore, the results indicate that the positive emotion joy, in turn, promoted prosocial behaviors toward other people; that is, the mediating effect of joy between the intervention program and prosociality was confirmed. In other words, the program promoted joy, which in turn promoted prosocial behaviors among the participating adolescents. It is possible that the positive emotional state of joy enabled greater openness to the needs of others, thus promoting prosociality. On the other hand, it is likely that joy made it possible to trust others, a fundamental pillar of social life and an expression of social relatedness to others (Caprara et al., 2013; Gerbino et al., 2016).

The results indicated that the program was effective for promoting the positive emotion of gratitude toward others among the adolescents who participated in the intervention. By carrying out the different activities proposed by the program, adolescents were able to experience the psychological benefits of expressing gratitude to others, as suggested by previous research (Emmons et al., 2003; Oros, 2014). The adolescents who participated in the Hero Program affirmed that the activities made them more aware of the help they receive from others. This was evidenced by the participants' statements that after the end of the intervention, “It has given me more of a desire to return favors,” “I called my teacher and thanked him/her for his/her patience during the virtual classes,” and “I was able to recognize the efforts of my parents during all these months” of isolation. Furthermore, our results indicated that the positive state of gratitude led the adolescents to be more prosocial toward others. That is, the mediating effect of gratitude on the promotion of prosociality was tested, and the results went in the same direction as previous research showing that a genuine feeling of gratitude promotes prosocial behaviors, since the grateful person seeks to reward the actions of the benefactor (Emmons et al., 2003; Oros, 2014). It is likely that the adolescents who developed higher levels of gratitude in the context of the intervention were better able to perceive the social support provided by others (McCullough et al., 2002; You et al., 2020), which favored solidarity behavior.

The program also proved to be effective for promoting *serenity*, which encourages peace and relaxation. It is likely that the program's facilitation of serenity in the adolescents enabled them to better cope with the social isolation they were experiencing, distancing themselves from thoughts that can generate concern or anxiety. This point was exemplified in the statements of the Colombian adolescents who participated in the Hero Program: “I really liked the calming exercises; I felt relaxed”; “After doing the activities, I was very calm at home.” Similarly, the results indicated that serenity in turn promoted prosocial actions toward others. The fact that the program promoted relaxation and tranquility may have fostered harmonious interpersonal relationships that favored the emergence of prosocial behaviors, despite the adverse events that the adolescents may have been experiencing.

Another positive emotion that favored the effects of the Hero Program in Colombian adolescents during isolation was the perception of *personal satisfaction*. It is evident that the program contributed to the process of valuing and promoting the self among the adolescents, encouraging them to recognize the merits of their actions (Diener and Larsen, 1993; Oros, 2014). Additionally, the strengthening of personal satisfaction in adolescents contributed to their development of prosocial behaviors. It is likely that the program facilitated greater self-acceptance and, therefore, greater acceptance of others (Diener and Larsen, 1993), thus favoring prosocial behavior.

Contrary to expectations, the results indicated that the program was not effective for promoting *sympathy* among the adolescents who participated in the intervention. Sympathy is understood as the affective component of empathy, which involves tuning into the emotions of the other or sharing the same emotional tone. It is likely that these findings can be explained by deeply rooted cultural patterns in Colombia, where the upbringing of children and adolescents aims to prioritize their emotions over those of others. This was reflected in the comments made by the adolescents at the end of the intervention, expressing their concern about being “invaded by the feelings of others” or parenting patterns that emphasized the need to “be strong and not get involved with what others feel.” These expressions, while denoting a certain capacity for recognizing the emotions of others, indicate a position of distancing oneself from the emotions of others out of fear of experiencing emotional distress. This could also be linked to the social and political context in which Colombian adolescents live. This context arises from two aspects, the historical and the current: historically, it arises from more than six decades of structural and political violence, and currently, it relates to the extreme social isolation caused by the COVID-19 pandemic. Research carried out in the Colombian context reports that children and adolescents who have experienced violence in environments of social vulnerability show weaker relationships between sympathy and behaviors associated with collaboration and helping others (Gómez-Tabares and Narvaéz Marín, 2020). Additionally, Moffitt (1993) states that it takes time for adolescents to accept the emotions they have toward others because they fear being ridiculed and not accepted by their peer group or by people who are not part of their immediate environment (Luengo Kanacri et al., 2020). Hence, it is likely that these adolescents interpret sympathy as a weakness of character that is not necessary for social life or the emergence of prosocial behavior. However, this interpretation should be made with care because there was a trend toward the Hero Program's promoting sympathy when compared to the pre-test evaluation. Consequently, another likely explanation is that the study failed to detect the effect of fostering sympathy vs. the pre-test evaluation due to insufficient statistical power.

Finally, the Hero Program proved to be effective for directly promoting prosocial behavior in the Colombian context of social isolation. This was observed through a statistically significant difference in the pre- and post-test evaluations between the control and intervention groups. The structure of the intervention brought adolescents closer to social situations to which isolation had limited their access (Alomo et al., 2020; Rodríguez-Morales et al., 2020), promoting the importance of closeness and solidarity with others within the complexity of social confinement (Ali et al., 2019; Espada et al., 2020; Stankovska et al., 2020). In this sense, the positive effect of the Hero Program may be linked to the fact that preventive social isolation—characterized by boredom, demotivation and irritability in young people (Imran et al., 2020)—was modified by the program's activities, which guided adolescents to be more open to the needs of others. This result is also confirmed by the participants' comments after the intervention, such as “I will try to help people more, especially in this difficult time of COVID”; “I think it is important to think about others and be able to help them… I truly liked that aspect of the program”; “I would be happy to help someone when they are sick.” These results are consistent with previous research showing the effectiveness of the Hero Program for mitigating the adverse effects generated by isolation in other Latin American countries by promoting prosociality (Mesurado et al., 2020).

In summary, this study concludes that the Hero Program was effective for promoting four positive emotions (joy, gratitude, serenity and personal satisfaction) and that these emotions predispose Colombian adolescents to act prosocially. Additionally, the program was effective for directly promoting prosociality in adolescents during isolation. This study is particularly relevant because it is necessary to develop interventions with proven efficacy to counteract the trauma produced by social isolation in young people around the world.

### Limitations of the Study and Future Directions

An important limitation of this study is that it did not include a follow-up evaluation of the measurements of positive emotions and prosociality to verify whether the effect of the program remains stable over time. Furthermore, given that we did not find an effect of the program on the promotion of sympathy, despite its effects on the other positive emotions evaluated and prosociality, it would be interesting for future studies to analyze whether the Hero Program is effective in promoting the cognitive aspect of empathy, which is linked to taking perspective or understanding the emotional tone of others. Finally, it would be interesting in future studies to implement the program in other contexts of economic, social or cultural vulnerability to analyze its effectiveness in diverse contexts.

## Data Availability Statement

The datasets presented in this study can be found in online repositories. The names of the repository/repositories and accession number(s) can be found below: https://riu.austral.edu.ar/handle/123456789/885.

## Ethics Statement

The research protocol of this study was approved by the Comité Institucional de Evaluación of the Facultad de Ciencias Biomédicas of Universidad Austral [CIE N° P 20-058], and it was ratified by the ethics committee of the Ibero-American University Corporation.

## Author Contributions

BM, SR, MT and CV made substantial contributions to the conception of the study, the acquisition, analysis, interpretation of the research data, and preparation of the manuscript for publication. All authors contributed to the article and approved the submitted version.

## Conflict of Interest

The authors declare that the research was conducted in the absence of any commercial or financial relationships that could be construed as a potential conflict of interest.

## Publisher's Note

All claims expressed in this article are solely those of the authors and do not necessarily represent those of their affiliated organizations, or those of the publisher, the editors and the reviewers. Any product that may be evaluated in this article, or claim that may be made by its manufacturer, is not guaranteed or endorsed by the publisher.
